# Combining kinetics and in silico approaches to evaluate bromhexine as an anti-pancreatic lipase agent for obesity management

**DOI:** 10.1038/s41598-025-02625-4

**Published:** 2025-05-26

**Authors:** Asma Gholami, Dariush Minai-Tehrani, Leif A. Eriksson

**Affiliations:** 1 Department of Chemistry and Molecular Biology, University of Gothenburg, 405 30 Göteborg, Sweden; 2 Bioresearch Lab, Faculty of Life Sciences and Biotechnology, Shahid Beheshti University, Tehran, Iran

**Keywords:** Lipase, Obesity, Bromhexine, Molecular docking, Inhibition mechanism, Computational biology and bioinformatics, Drug discovery, Molecular dynamics

## Abstract

Industrialization, fast food intake and reduced physical activity, mainly in developed countries, exacerbate obesity and make it a major lifestyle disorder. A promising strategy for developing effective anti-obesity agents is to inhibit pancreatic lipase, thereby reducing lipid absorption. Currently, the only clinically approved pharmacological agent for pancreatic lipase inhibition is Orlistat. However, its undesirable gastrointestinal side effects have prompted the search for more effective and potent drugs. This study investigates the inhibitory mechanism of Bromhexine, a mucolytic drug, on pancreatic lipase using Lineweaver–Burk plot analysis and molecular docking, along with simulations, and compares its efficacy to that of the Food and Drug Administration (FDA) approved drug Orlistat. Kinetic analysis indicates that Bromhexine exhibits mixed inhibition of pancreatic lipase, with IC_50_ and K_i_ values of 360 µM and 450 µM, respectively, which are comparable to those of Orlistat. Molecular docking confirms that Bromhexine interacts with the His263 residue in the enzyme’s active site through hydrogen bonding, similar to Orlistat, thereby reducing the enzyme’s affinity for its natural substrate. Binding pose metadynamics (BPMD) simulations further supports the stability of Bromhexine’s interactions. Collectively, our findings suggest that Bromhexine displays potent pancreatic lipase (PL) inhibition activity and could serve as a potential candidate in weight management as demonstrated by both in silico and in vitro analyses. However, further investigations, including structure-activity relationship (SAR) analyses and in vivo studies, are necessary to confirm its clinical potential as a pancreatic lipase inhibitor.

**Supplementary Information:**

The online version contains supplementary material available at 10.1038/s41598-025-02625-4.

## Introduction

Urbanization and sedentary lifestyles have significantly increased obesity rates, particularly in recent years^1^. It contributes to the escalation of various health hazards, including metabolic syndrome, diabetes mellitus, gynecological abnormalities, stroke, nonalcoholic fatty liver disease, and certain cancers^2,3^.

Studies show that various factors, such as genetic, environmental, hormonal and dietary influences, control body weight^4^. Consequently, the life-threatening risks associated with obesity necessitate the involvement of scientists in its prevention and treatment, making it a critical health priority^2^. One strategy for treating obesity is the development of enzyme inhibitors that do not change major mechanisms in the gastrointestinal tract but decrease the digestion and absorption of dietary lipids^2,5^.

Pancreatic lipase (PL) is a key enzyme secreted by the pancreas, that is responsible for hydrolyzing 50–70% of dietary fat, making it a critical target for fat absorption. Because of its essential role in lipid metabolism, PL has become a promising target for the development of drugs aimed at treating obesity and other metabolic disorders. Characterizing and identifying inhibitors of this enzyme could lead to effective therapeutic interventions^1,3^.

Orlistat and Sibutramine are two drugs that have been approved for long-term clinical use by the Food and Drug Administration (FDA)^6^. Orlistat reversibly inactivates gastric and pancreatic lipases. When lipase is inhibited, the hydrolysis of triglycerides is blocked, preventing the absorption of free fatty acids^1^. However, Orlistat can cause adverse side effects such as steatorrhea, flatulence, and fecal incontinence, which often lead specialists to refrain from prescribing this drug^7,8^.

Sibutramine, on the other hand, affects both the noradrenergic and serotonergic (5HT) pathways in the hypothalamus, which are involved in energy balance. It inhibits the reuptake of serotonin and noradrenaline released from hypothalamic neurons, which are key to its anti-obesity effects^9^. However, Sibutramine can cause several unpleasant side effects, including dry mouth, headache, insomnia, asthenia, constipation, and, in some cases, amnesia. Furthermore, due to a significantly increased risk of cardiovascular events, the use of Sibutramine was withdrawn by the FDA in the United States of America and the European Medicines Agency in 2010^9,10^. Therefore, the search for new, safe medications with fewer adverse effects for treating obesity remains a critical area of research and is a prominent focus in the field.

**Fig. 1:**
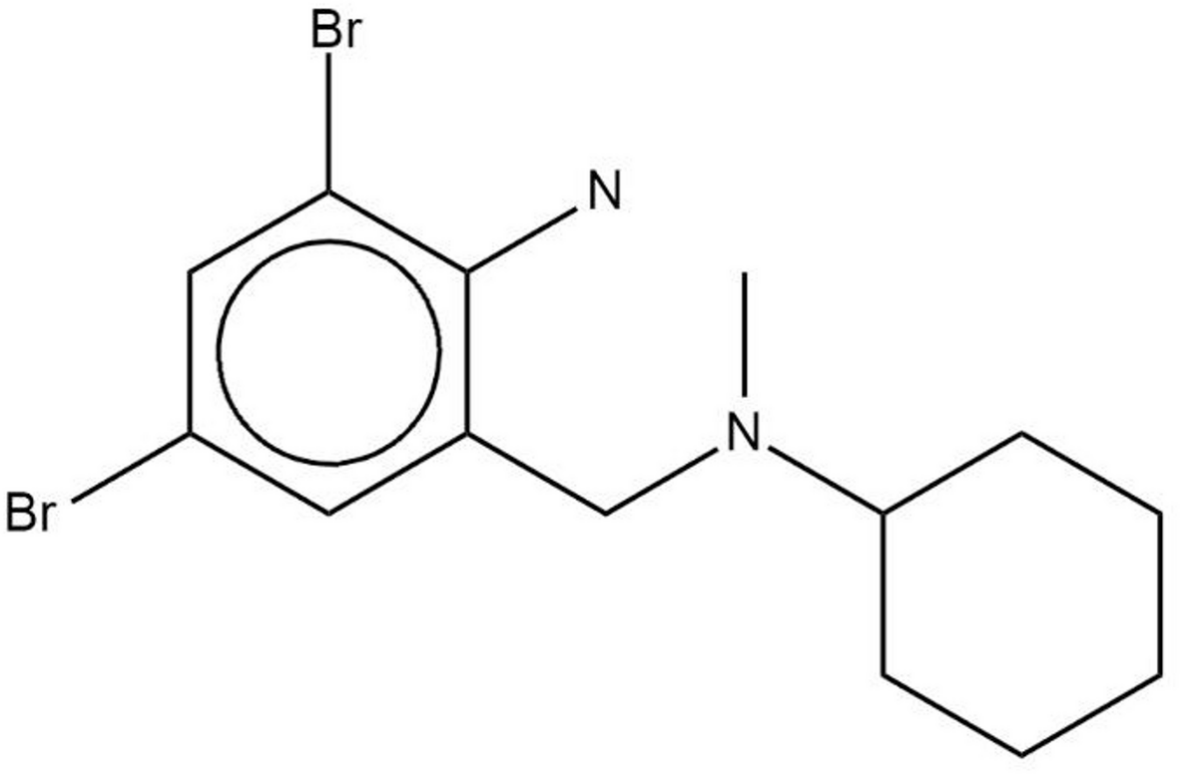
Chemical structure of bromhexine.

Bromhexine (Fig. 1) is commonly prescribed as a mucolytic agent to treat excessive mucus secretion in the respiratory tract^11,12^. In addition, Bromhexine hydrochloride has been shown to reduce mortality in patients with COVID-19^13^. In this case, for the patients treated with Bromhexine the severity of the disease was milder, and the need for Intensive Care Unit (ICU) transfer, intubation, and mechanical ventilation was significantly reduced^14^.

Our previous studies demonstrated that Bromhexine can inhibit lipase^15^, a major virulence factor in *Pseudomonas aeruginosa* (as *prokaryotic* model organism, and multi-resistant bacteria). This bacterium often infects patients with cystic fibrosis, burn injuries, or compromised immune systems^16,17^. The half maximal inhibitory concentration (IC_50_) and inhibition constant (K_i_) values of Bromhexine were calculated to be 49 µM and 20 µM, respectively^15^.

The current study focuses on investigating the inhibitory effect and binding characteristics of Bromhexine as a potential PL inhibitor (as *eukaryotic* model organism), as compared to Orlistat. Various methods, including molecular docking and molecular dynamics simulations, were employed. Given that several anti-obesity medications have been withdrawn or found to have serious side effects, Bromhexine represents a promising candidate as a novel PL inhibitor. The study also examines the kinetic parameters of inhibition and the nature of the inhibitor’s binding to the enzyme. The findings indicate that Bromhexine could offer a new approach to inhibiting fat absorption, potentially with fewer side effects.

## Materials and methods

### In silico studies

#### Protein preparation

The holoenzyme of the lipase protein in its open structure with methoxy undecyl phosphonic acid co-crystallized (PDB ID: 1LPB)^18^ was downloaded from the Protein Data Bank (PDB) (http://www.rcsb.org). The protein was prepared and refined using the Protein Preparation Wizard in Maestro (Schrödinger 2024-1, www.schrodinger.com). Bond orders were assigned during the preprocessing stage of the crystal structure. After retrieving missing loops or side chains, all water molecules situated more than 3.0 Å from the ligand were removed. Protein hydrogen bond assignments were optimized, and protonation states at pH 7 were determined using PROPKA^19^. Finally, restrained minimization was performed with the OPLS4 force field^20^, applying an RMSD convergence threshold of 3.0 Å for the heavy atoms.

#### Ligand preparation and docking

Ligand structures for Bromhexine and Orlistat were downloaded from the PubChem database (https://pubchem.ncbi.nlm.nih.gov) and imported into Maestro Schrödinger using LigPrep. The Epik module^21^ was employed to assess possible protonation states at physiological pH (7.0 ± 2.0), and the OPLS4 force field was chosen for optimization.

Protein and ligand docking studies were conducted using the Glide Ligand Docking module^22^ within the Schrödinger Suite. This module utilizes a grid-based method that evaluates energetics and generates scores based on the formation of favorable interactions between the molecule and the protein. Rigid docking was employed to ensure that the protein structure remained fixed during the docking process. The docking grid was defined with coordinates X: 4.37, Y: 28.09, Z: 48.8, specifically targeting the catalytic triad residues Ser152, Asp176, and His263.

The prepared ligands Bromhexine (inhibitor under study) and Orlistat (reference inhibitor) were docked into the grid-enclosed active pocket of the protein using the Extra Precision (XP) mode. Protein-ligand complex interactions were assessed based on the quality of geometric contacts and their associated energy^22^.

#### Molecular dynamics (MD) simulations

The stabilities of the ligand–protein complexes were investigated through 200 ns molecular dynamics (MD) simulations conducted in triplicate, using an NPT ensemble and the OPLS4 force field with the Desmond MD engine^23^ implemented in Schrödinger (Schrödinger 2024-1, www.schrodinger.com). Water molecules were modeled using the TIP3P force field^24^. Periodic boundary conditions were applied with a 10 Å water buffer around the protein in a cuboid simulation box. The system was neutralized by adding an appropriate number of counterions (i.e., Na^+^/Cl^−^) and adjusted to a salt concentration of 150 mM to mimic physiological conditions. Temperature (300 K) and pressure (1 atm) were controlled using the Nose–Hoover thermostat (1 ps relaxation time) and the Martyna–Tobias–Klein barostat^25^ with isotropic coupling, respectively. Electrostatic forces were handled using particle-mesh Ewald (PME) summation^26^ with a cut-off of 9 Å for both electrostatic and van der Waals interactions.

The relaxation protocol comprised several steps:

1. NVT Brownian Dynamics with restraints on solute heavy atoms at T = 10 K for 100 ps,
2. NVT MD simulation at T = 10 K with restraints on solute heavy atoms for 12 ps,
3. NPT MD simulation at T = 10 K with restraints on solute heavy atoms for 12 ps,
4. NPT MD simulation at T = 300 K with restraints on solute heavy atoms for 12 ps, and.
5. NPT MD simulation at T = 300 K without restraints for 24 ps.

#### Clustering

From the MD trajectories, clustering of the resulting structures was performed to group similar molecular conformations into distinct sets, ensuring that the structures within each cluster are more similar to each other than to those in other clusters. This approach provides a refined view of how a given molecule samples the conformational space and allows for direct characterization of the distinct conformational sub-states visited during the MD simulation. To achieve this, the Desmond Trajectory Clustering tool in Maestro (Schrödinger 2024-1, www.schrodinger.com) was utilized. Protein backbone atoms were specified in the Atom Specification Language (ASL) dialogue box, with the initial number of clusters set to 3. After running the clustering tool, the structures of each cluster that exhibited the largest number of similar conformations were selected for further analysis. Specifically, these selected complexes were then evaluated to identify those conformers with the lowest energies and highest stabilities.

#### Binding pose metadynamics (BPMD)

Binding Pose Metadynamics (BPMD) simulations were conducted using a biasing force to explore the stability of ligands within the binding pocket of the receptor. Weaker ligands are expected to exhibit higher fluctuations and larger RMSD values compared to those that are stably bound^27^. The MD clustering in the previous stage eliminated any unfavorable contacts and enabled the selection of the most suitable starting structures. Subsequently, BPMD simulations were carried out using Schrödinger Maestro version 2024-1. For each system, 10 independent metadynamics simulations of 10 ns each were performed, with the Root-Mean-Square Deviation (RMSD) of the ligand heavy atoms relative to their starting position used as the Collective Variable (CV).

### In vitro studies

#### Materials

The chemicals used for culture media and buffer preparation were of reagent grade. Para-nitrophenyl palmitate (pNPP) was purchased from Sigma Aldrich, Merck KGaA, Darmstadt, Germany (https://www.sigmaaldrich.com). Bromhexine hydrochloride of pharmaceutical grade was sourced from Chemidarou (https://www.chemidarou.com).

#### Sample collection

Five Sprague–Dawley rats (*Rattus norvegicus*), aged 8–10 weeks and weighing 200–250 g, were included in this study. The rats were purchased post-mortem from the Animal Research and Service Centre at Shahid Beheshti University; no live animal experimentation or euthanasia was performed as part of this study. For the lipase assay, the pancreas of each rat was dissected, and the enzyme was extracted. The study protocol was approved by the Ethics Committee of Shahid Beheshti University (SBU) in Tehran, Iran, with approval number IR.SBU.REC.1398.042. All experimental methods and protocols are compliant with the relevant ARRIVE guidelines. The study was conducted in accordance with relevant guidelines and regulations.

#### Enzyme extraction

After removing other debris from the pancreatic tissue, the organ was washed three times with an isotonic solution (0.15 M NaCl). Subsequently, the tissue was homogenized using a 0.1 M phosphate buffer at pH 7. Each homogenate was centrifuged at 8000 × g for 4 min to pellet the intact cells. The supernatant was then collected for enzyme assay and subsequent analysis.

#### Enzyme assay

The buffer for the enzyme assay was prepared by adding varying concentrations (0.02–0.5 mM) of p-nitrophenyl palmitate (pNPP), which is soluble in isopropanol, to a 0.1 M Tris buffer at pH 8 containing 0.1% (v/v) Tween 80. The reaction was initiated by adding 50 µL of the enzyme supernatant to the working buffer in a test tube, with the final volume in each test tube maintained at 2 mL. Lipase catalytic activity was monitored by tracking the increase in yellow color of the product, p-nitrophenol, with an absorption peak at 410 nm using a UV-Visible spectrophotometer^15,28^. All assays were conducted at room temperature (25–28 °C) and performed in triplicate. The enzyme activity was measured in the absence and presence of Bromhexine at concentrations ranging from 0 to 0.3 mM. The assay was conducted over a 5-minute period, which was sufficient for the enzyme activity to reach a plateau, indicating substrate depletion.

#### Kinetic analysis of Inhibition and the half maximal inhibitory concentration (IC_50_)

By adding different concentrations of Bromhexine and pNPP to PL, the inhibition type of PL by Bromhexine was investigated. The kinetic parameters were evaluated using Lineweaver–Burk plot^29^. The IC_50_ value was furthermore determined from the data at varying concentrations of Bromhexine and compared with literature data for Orlistat.

## Results and discussion

### In silico studies

#### Molecular docking

The mechanism of interaction between the receptor and ligand, along with the energetics, can be investigated by directly observing the binding conformation and binding site of the receptor and ligand^30^. Based on our previous studies on lipase in *P. aeruginosa* and other prokaryotic/eukaryotic microorganisms, the sequence identity between our reference protein (*P. aeruginosa*) and human pancreatic lipase is only 9%, indicating that the enzyme is less conserved across species. However, a closer look at the aligned sequences reveals that the active site serine residue is conserved across all organisms, underscoring the importance of this amino acid in the catalytic site^11^.

**Fig. 2:**
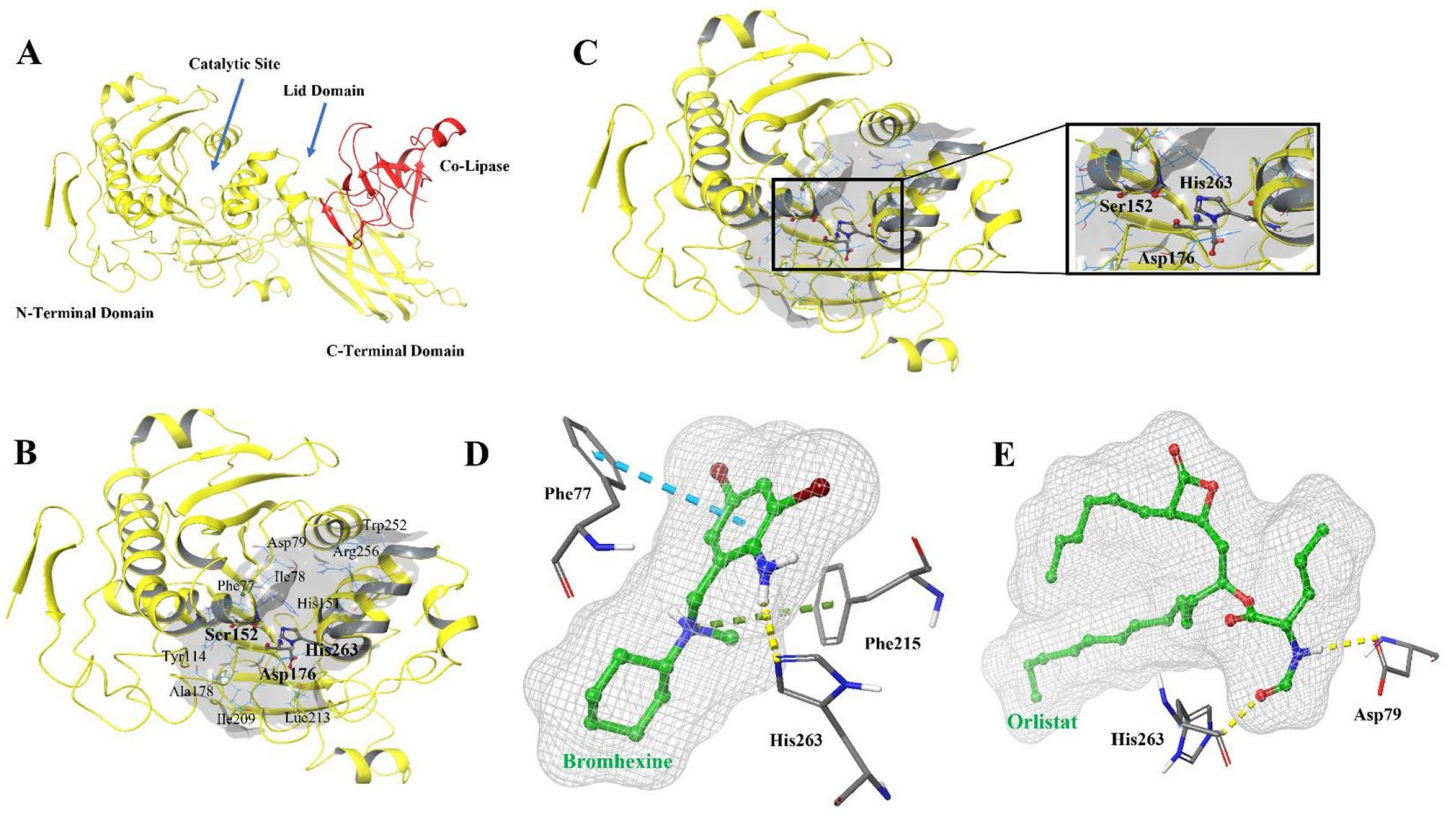
(**A**) Structure of the PL protein with Co-lipase (red) bound. (**B**) Structure of the PL (yellow) catalytic region (grey surface), the catalytic triad is represented in stick model, and several peripheral amino acids involved in inhibition, positioned in close proximity to the active site pocket, are also labelled. (**C**) Zoomed-in 3D structure of PL, highlighting the active site residues in the catalytic triad. (**D**,**E**) Atomic contact interaction patterns for Bromhexine and Orlistat. Different types of interactions are represented by color-dashed lines: blue for π–π stacking, yellow for hydrogen bonds, and green for π-cation interaction. All images generated using Schrödinger Maestro 2024-1.

In this study, Bromhexine and Orlistat (as reference compound) were docked to the crystal structure of the pancreatic lipase-colipase complex (PDB ID: 1LPB, 2.46 Å resolution) The activity of the enzyme is obtained through the catalytic triad Ser152, Asp176, and His263. The most significant residue for lipolysis activity is Ser152. In the presence of bile, colipase, or phospholipids, the C-terminus is the site of interaction (Fig. 2A). Activation of the enzyme occurs at water-lipid interfaces to interact with water-insoluble substrates and hydrolyze them. When mixed bile salt phospholipid micelles are present near the lid site, there is a conformational change whereby the enzyme opens and the two domains rotate slightly, so the N-terminal domain instead interacts with colipase^31^. A closer look at the structure of the protein reveals that certain peripheral amino acids located near the active site are critical for the inhibition of PL. Among these, Tyr114, Asp79, and Phe77 are particularly important (Fig. 2B). These amino acids, although not directly involved in the catalytic process, play a significant role in stabilizing the interaction between the inhibitor and the enzyme^32^.

The active site of the lipase holoenzyme (Fig. 2C) was selected for docking. The Glide docking scores and calculations of free energies of binding were performed using the lowest energy structures of the enzyme obtained after clustering of the MD simulation trajectories. Table 1 shows the docking scores, and free energies of binding obtained using MMGBSA. The docking score and MMGBSA free energy of binding for Bromhexine were − 6.517 kcal/mol and − 45.32 kcal/mol, respectively (Table 1).

Despite having a lower docking score, the MMGBSA result for Orlistat shown in Table 1 (− 76.50 kcal/mol) indicates a stronger binding affinity to PL. Figure 2D shows that Bromhexine docks partly into the hydrophobic cavity at the active site of PL. The main residue interacting with Bromhexine is His263 through hydrogen bonding. In addition, the ligand forms π–π stacking interaction with the aromatic ring of Phe77 and π-cation interaction with Phe215. Our observations that the interaction occurs with His263 in the binding pocket, along with the residues at the peripheral site such as Phe77, indicates that Bromhexine binds to PL through mixed inhibition. In contrast, Orlistat (Fig. 2E) forms two hydrogen bonds with His263 (in the catalytic site) and Asp79, thus making it more potent towards PL.

As previously pointed out, hydrogen bonding plays a significant role in the interaction between the ligand and PL^33^. From the current findings, Bromhexine interacts with the His263 residue in the active site of PL and also blocks the substrate channel, resulting in the inhibition of PL activity.

**Table 1:** Docking score values and free energies of binding (MMGBSA) for each compound, obtained from the virtual screening.

Ligand	Docking score (kcal/mol)	MMGBSA (kcal/mol)
Bromhexine	− 6.517	− 45.32
Orlistat	− 5.220	− 76.50

#### Molecular dynamics simulations

**Fig. 3:**
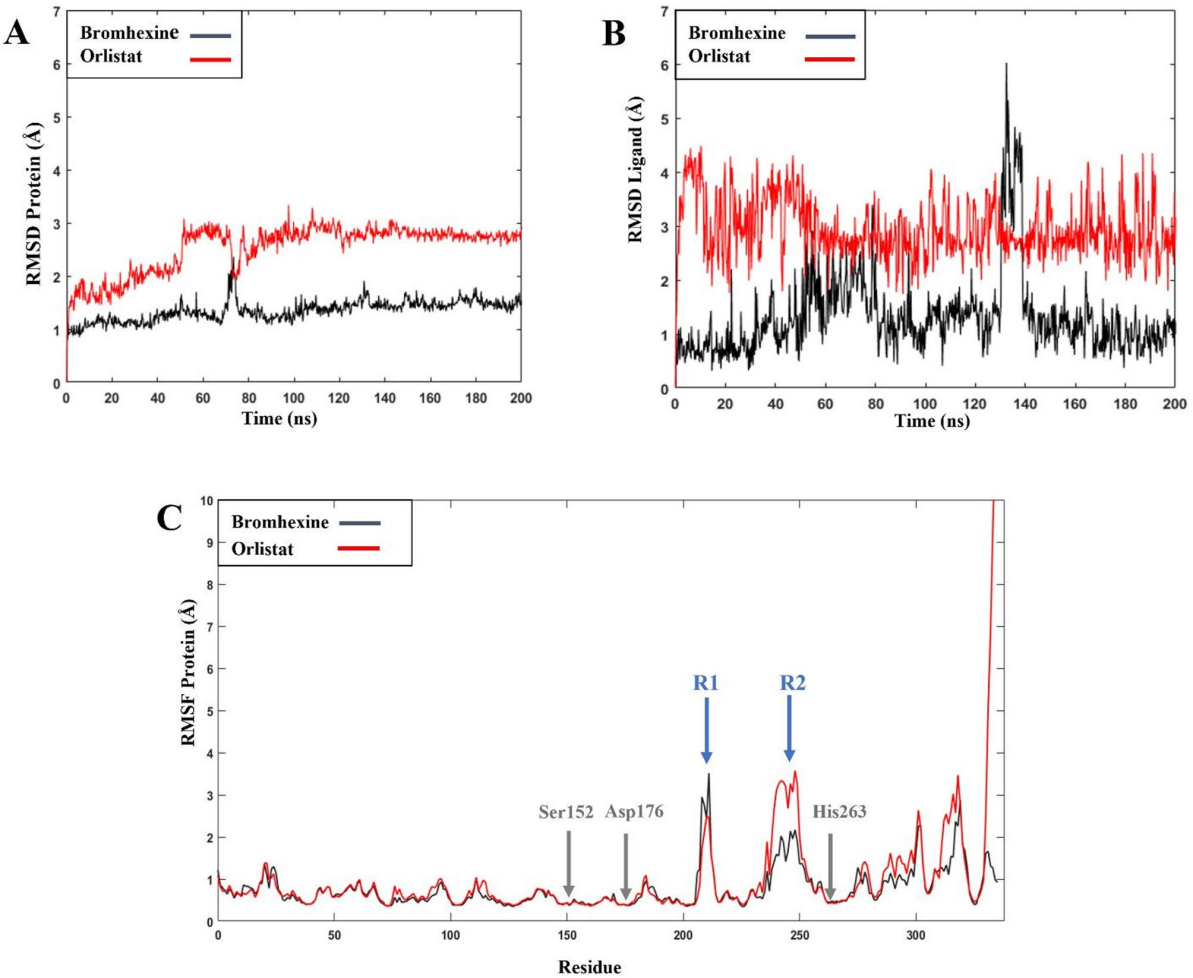
(**A**,**B**) Root-mean-square deviation (RMSD) values for the protein and ligand during one of the triplicate 200 ns MD simulations. (**C**) Root-mean-square fluctuations (RMSF) of PL during 200 ns MD simulations in complex with Bromhexine and Orlistat. Gray arrows correspond to the location of the catalytic residues. Blue arrows indicate the parts of the protein that undergo more movement during the simulation (see text for details).

The stability of the complex structure can be analyzed by evaluating the RMSD during the simulation trajectory. As shown in Fig. 3A, the RMSD for PL (protein) stabilized after 70 ns for both ligands in simulation replica 1, with no changes observed thereafter. The average RMSD of the protein in the presence of Bromhexine and Orlistat was estimated at 1.343 Å and 2.527 Å, respectively (Fig. 3A). This consistency further underscores the structural integrity of the protein during the simulations and highlights that ligand binding, particularly with Bromhexine, did not induce any destabilizing conformational changes. Analysis of the RMSD for the protein in each replica (Figs. S1 and S2) also indicates that the protein did not undergo any significant structural changes, despite a slight increase in RMSD values for Orlistat in replica 3.

The RMSD for Orlistat as ligand (Fig. 3B) has an average RMSD of 2.943 Å. It appears that the compound preserves its binding interactions and remains tightly bound to the catalytic site. No significant fluctuations were observed throughout the simulation periods, implying that the binding of the ligand to the active site of PL is very stable and strong. This data is confirmed by the average values in each replica for this ligand (Table S2 and Fig. S2).

The protein-ligand interactions analyzed using data from one of the 200 ns MDs trajectories (Figure S3A) show that Orlistat forms key interactions primarily hydrogen bonds, with multiple residues. Notably, a hydrogen bond with Ser152 is present during 55% of the simulation, showing its significance in stabilizing the ligand–protein complex and the importance of this amino acid as the main catalytic residue. Other interactions are observed with Asp79 and Phe77, at 53% and 16% duration in the simulations, respectively, which both involve hydrogen bonding. These interactions collectively contribute to the overall stability and binding affinity of the ligand in the active site.

Although the RMSD values for Bromhexine are significantly lower compared to Orlistat (Fig. 3B), with the average RMSD value 1.307 Å, a sudden sharp peak is observed during the 130–140 ns interval. Our observations for each replica (Fig. S1 and Table S1) suggest that these changes are due to the partial exposure of the benzene ring of the ligand to the solvent. However, for the remainder of the simulations in replica 1 and replica 3, equilibrium was reached after 140 ns, confirming that the ligand remained very stably bound to the binding pocket. The consistently lower RMSD values for Bromhexine, compared to Orlistat, highlight a more stable and robust interaction with PL and emphasize its potency as a viable alternative to Orlistat for obesity management.

Root mean square fluctuations (RMSF) of each amino acid in the protein was used to determine the flexibility of certain regions of the molecule (Selvan et al., 2010). Figure 3C shows the fluctuation of each amino acid in both the protein-ligand complexes in MD simulation replica 1. Apart from regions R1 and R2 (blue arrows), which exhibit higher mobility, the enzyme-inhibitor complexes are very stable. The locations of the three main residues, Ser152, Asp176, and His263, in the catalytic pocket are indicated by gray arrows. These residues remained highly stable during the MD simulations.

One of the regions with the highest variation corresponds to residues 208–213 (blue arrow, R1), which is not involved in the active site of the protein. The other region showing large movement is R2, which contains residues 240–253 and corresponds to a helix close to His263. However, a closer inspection revealed that these movements did not affect the active site of the protein for either ligand. Considering Fig. S1 and the average results (Table S1) for the RMSF in replica 1, some areas of the protein very close to the active site (Ser152 and Asp176) fluctuate the most during the simulation; however, there were no significant changes in the other two replicas.

The protein-ligand interactions for the Bromhexine complex were analyzed using data from a 200 ns MD trajectory (Fig. S3B), and show interactions between the brominated ligand and several residues in the protein binding pocket. A strong π-cation bond (91% presence) is formed between the amine group and Phe215, indicating its crucial role in stabilizing the ligand-protein complex. Additional π-cation interaction is seen between the same amine group and His263 with a weaker occupancy of 17%. Furthermore, water-mediated interactions are suggested with Thr255 through a hydrogen bond network, present during of 28% the simulation. The bromine atoms attached to the aromatic ring participate in a favorable interaction with Phe77 via π-π stacking, during 46% of the simulation. These results pin-point the same amino acids which are involved in docking pose (Fig. 2D). In comparison with Orlistat, which binds strongly to catalytic residue Ser152, Bromhexine hence also demonstrates the ability to bind the catalytic site, but primarily through an interaction with His263, suggesting an alternative binding mode.

The data obtained herein is in line with a recent study, in which a series of indole-TZD hybrids were synthesized and evaluated for their PL inhibitory potential^35^. It was suggested that two analogues (7k and 7 m) exhibited stable binding conformations throughout 20 ns simulations, with maximum RMSD deviations around 4–5 Å. The interactions of these molecules with 1LPB during the 20 ns MD run (using the GROMACS software) included one hydrogen bond and one π–π stacking interaction with Phe77 for both ligands. It was also observed that compound 7k formed a π–π stacking interaction with His263, as the main catalytic residue.

In comparison, we performed 200 ns MD simulations in triplicate, and the RMSD results for Bromhexine indicate a higher stability for this compound. In addition to the π–π stacking interaction with Phe77 seen both for Bromhexine and compound 7k^35^, it can be noted that Bromhexine also formed a strong hydrogen bond with the main catalytic residue, His263.

#### Hydrogen bonds

**Fig. 4:**
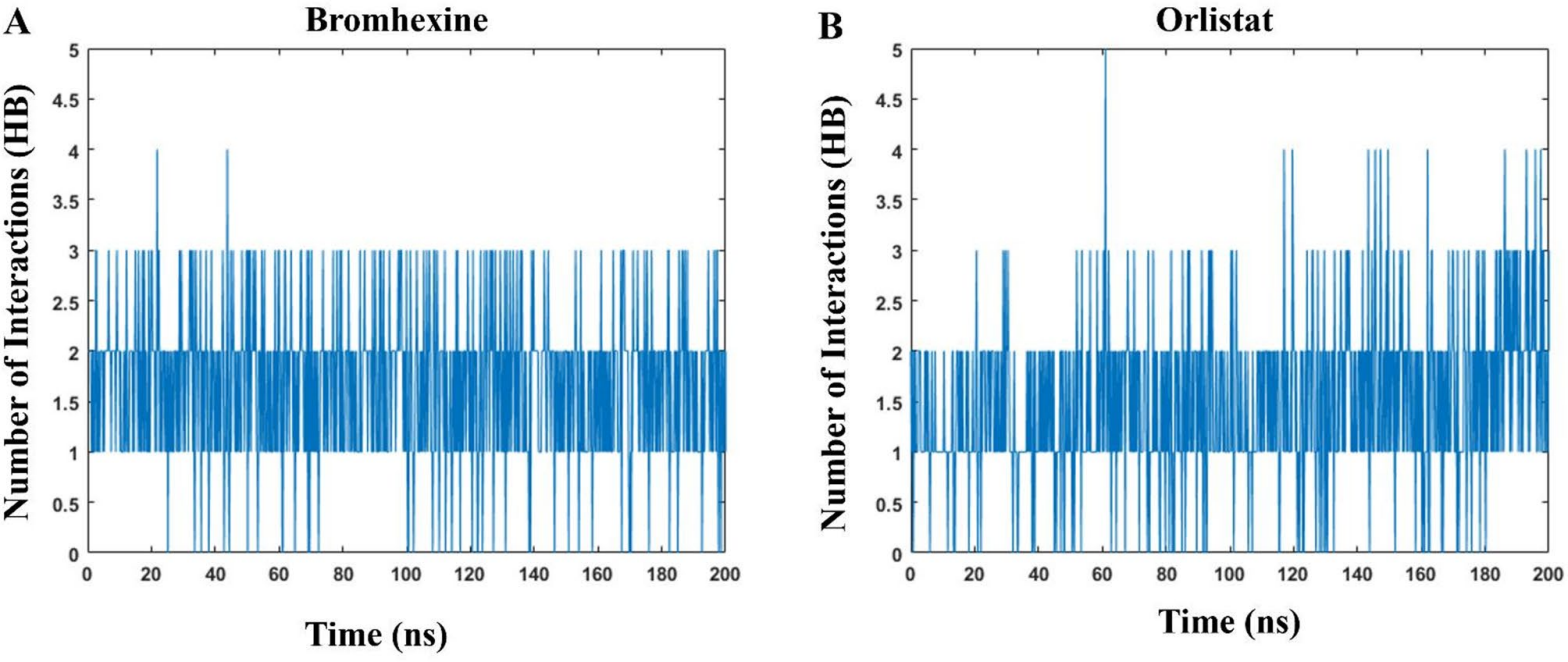
Graph of hydrogen bonds for Bromhexine and Orlistat during a 200 ns MD simulation.

To evaluate the robustness of the formed complexes, the number of hydrogen-bonded interactions was calculated during the simulations (Fig. 4). The average number of hydrogen bonds for Bromhexine and Orlistat were 1.29 and 1.88, respectively. These results confirm that Bromhexine is a promising binder, as its value is close to that of Orlistat.

#### Binding pose metadynamics (BPMD) simulations

BPMD simulations were conducted for both ligands. The stability of the pose was evaluated based on the PoseScore, which is the RMSD of the ligand relative to the initial coordinates of its heavy atoms. A PoseScore value of ≤ 2 Å is considered indicative of stable ligand binding in the pocket^27^.

**Fig. 5:**
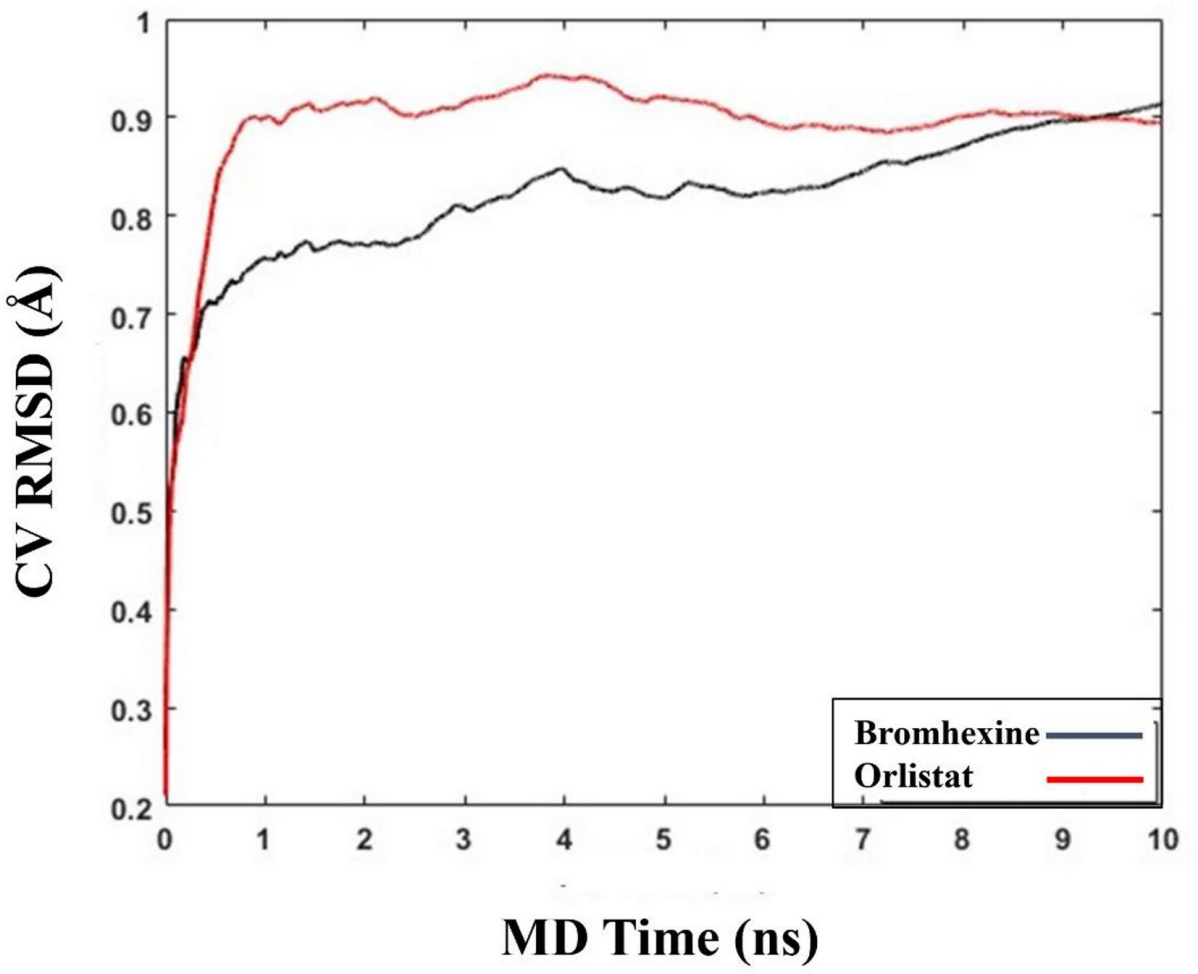
Graph of the RMSD estimate, averaged over 10 BPMD trials, for Bromhexine and Orlistat binding to PL, plotted against simulation time.

The BPMD analysis in Fig. 5 indicates that Orlistat maintains stable throughout the simulation time, suggesting a strong and consistent binding affinity. The average PoseScore for Orlistat was calculated to be 0.906 Å. Bromhexine exhibits minor variations toward the latter part of the BPMD simulation, which suggest some degree of flexibility in its binding pose. Despite this, the average PoseScore for Bromhexine was determined to be 0.895 Å, which, in comparing with the reference compound and established threshold values, indicates that Bromhexine maintains an overall stable binding interaction and remains a promising inhibitor to the enzyme.

### In vitro studies

#### Kinetic parameters

##### Type of inhibition

**Fig. 6:**
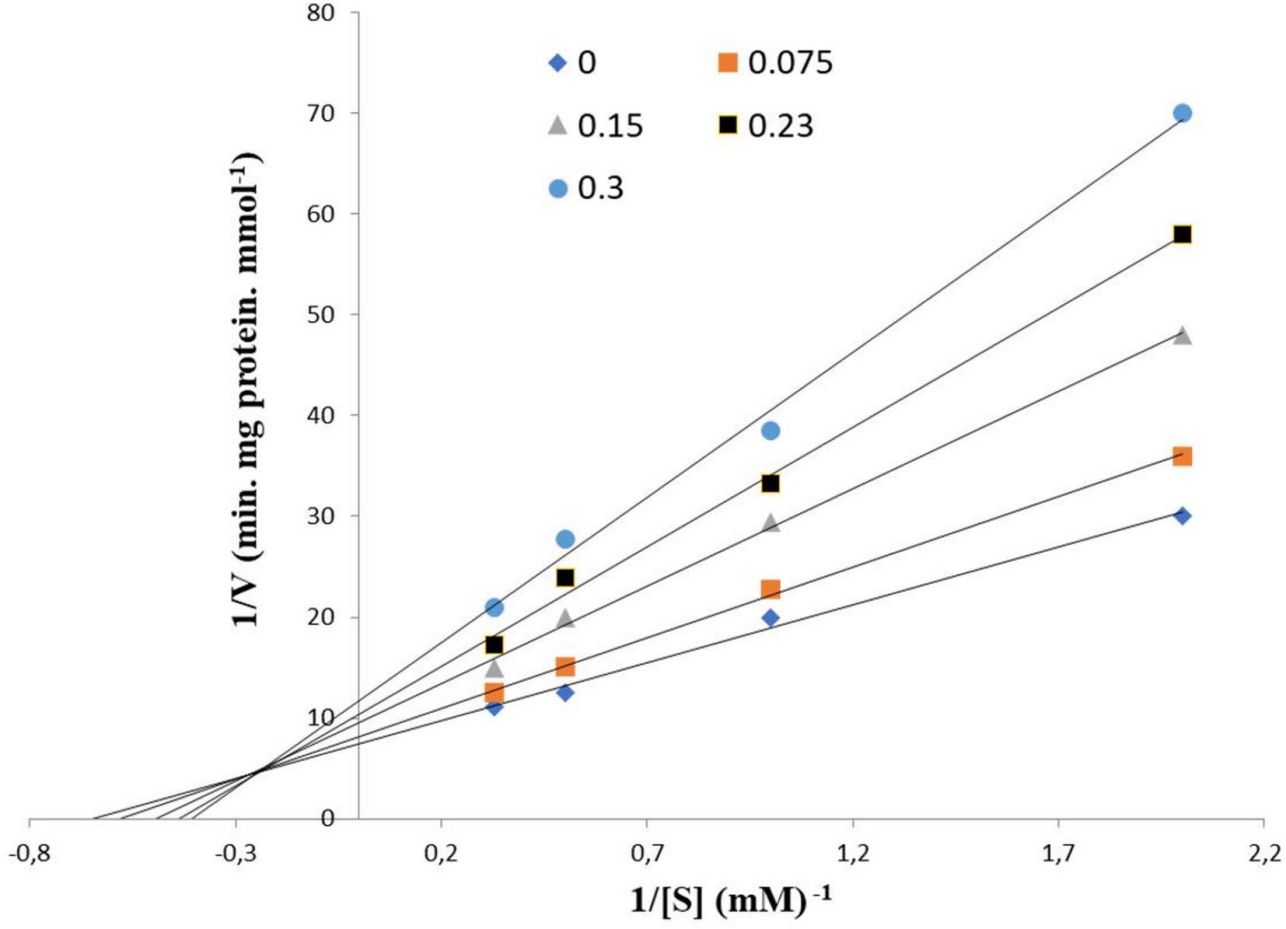
Lineweaver–Burk plot demonstrating mixed inhibition of PL by Bromhexine when present in the enzyme reaction medium. Bromhexine concentrations range from 0 to 0.3 mM.

Figure 6 displays the double reciprocal plot used to determine the kinetic parameters of the enzyme and the type of inhibition. It was found that Bromhexine inhibits PL in a mixed manner with respect to substrate concentration, as anticipated from the docking analyses, and a significant increase in K_m_ was observed. These results suggest that the drug can bind to both the free enzyme and the enzyme-substrate complex. As shown in Fig. 6, increasing the concentration of Bromhexine decreased the slope of the fitted lines. This pattern indicates that the inhibitory effect of the drug on PL is reversible and does not completely deactivate the enzyme.

A similar pattern was observed in a recent study where it was found that certain synthetic amino acid derivatives (PPC80, PPC82, PPC84) exhibited inhibitory effects on digestive enzymes, including pancreatic lipase, via competitive or mixed mechanisms^36^. Kinetic investigations have been conducted in numerous studies on various natural products and bioactive compounds with inhibitory activity against PL. For example, Kaempferol, a natural flavonoid compound found in cabbage, tea, broccoli, and other plants, was shown to synergistically inhibit PL activity in a competitive manner when combined with Orlistat^5^. Another study confirmed that Licochalcone A, isolated from the roots of *Glycyrrhiza uralensis*, exhibited inhibitory effects on pancreatic lipase, as demonstrated by Lineweaver–Burk plot analysis, and acted in a non-competitive manner^37^.

##### Inhibition constant (K_i_)

**Fig. 7:**
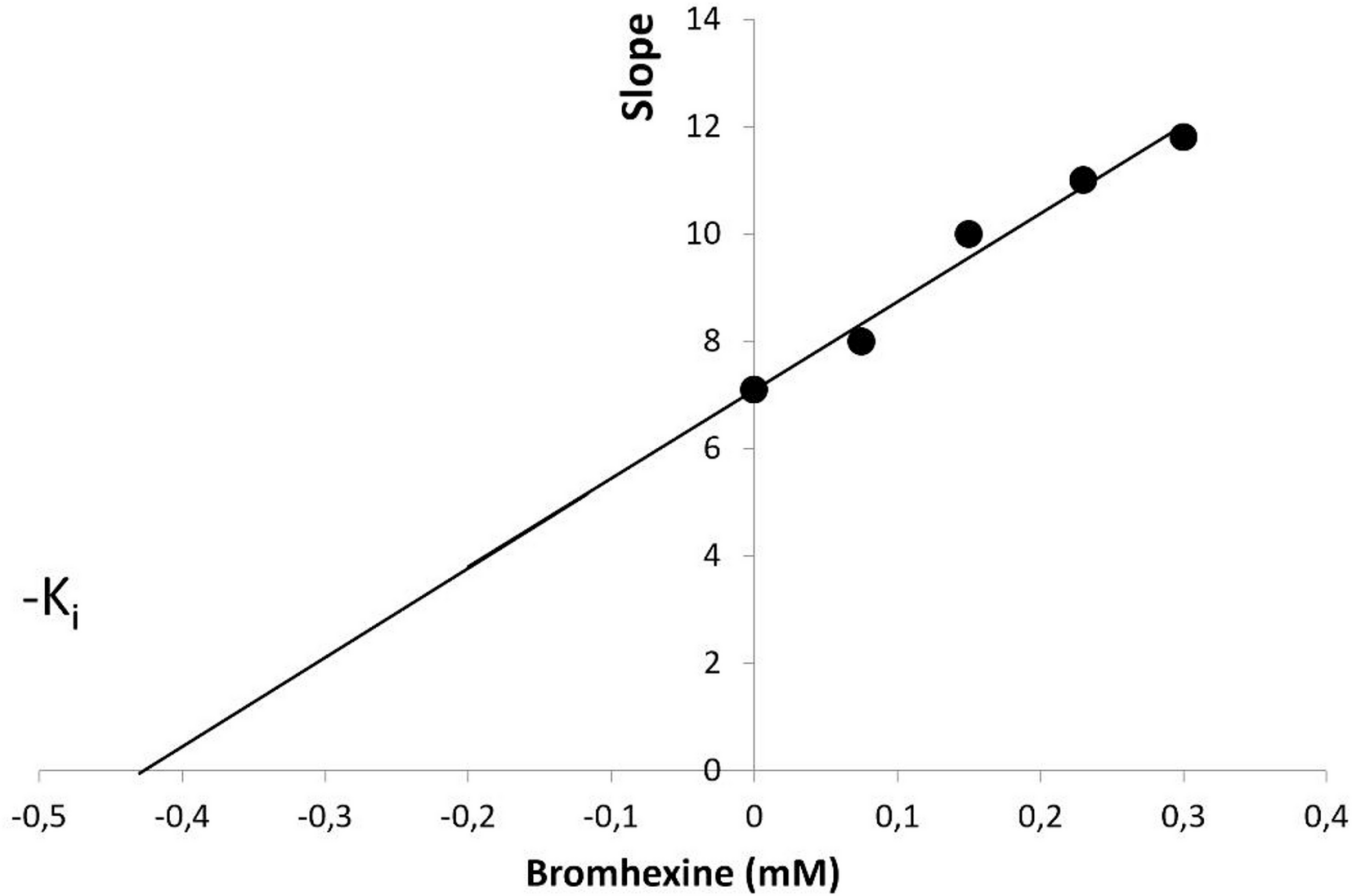
Secondary plot showing the K_i_ value of Bromhexine in PL. The slopes of the lines in the Lineweaver–Burk plot (Fig. 6) were used to calculate K_i_.

From the Lineweaver–Burk plot, a secondary plot shown in Fig. 7, was created to determine the K_i_ of the drug. The slopes from the primary plot were used for the secondary plot, with the x-intercept indicating the K_i_ value. The calculated K_i_ value was approximately 450 µM (0.45 mM), suggesting that Bromhexine exhibits a moderate affinity for PL.

There is a substantial need to study the safety and efficacy of medicinal plants and their compounds as therapeutic treatments for obesity, although only a few of these have progressed to drug development. For instance, Li et al. investigated the lipase inhibitory activity of Kaempferol, a natural anti-obesity supplement, using an in vitro assay and a UV-2450 spectrophotometer. Lipase activity was assessed by measuring the hydrolysis of p-nitrophenyl butyrate to p-nitrophenol. The K_i_ of Kaempferol was calculated to be 0.16 ± 0.07 mM (160 µM)^5^. Although Kaempferol appears to be a potent natural compound, the results indicated that combining Kaempferol with Orlistat produced a synergistic inhibition of PL at relatively low concentrations. Thus, while Kaempferol shows promise, it cannot fully replace Orlistat, and the irreversible side effects of Orlistat have not been completely mitigated.

##### Half maximal inhibitory concentration (IC_50_) determination

**Fig. 8:**
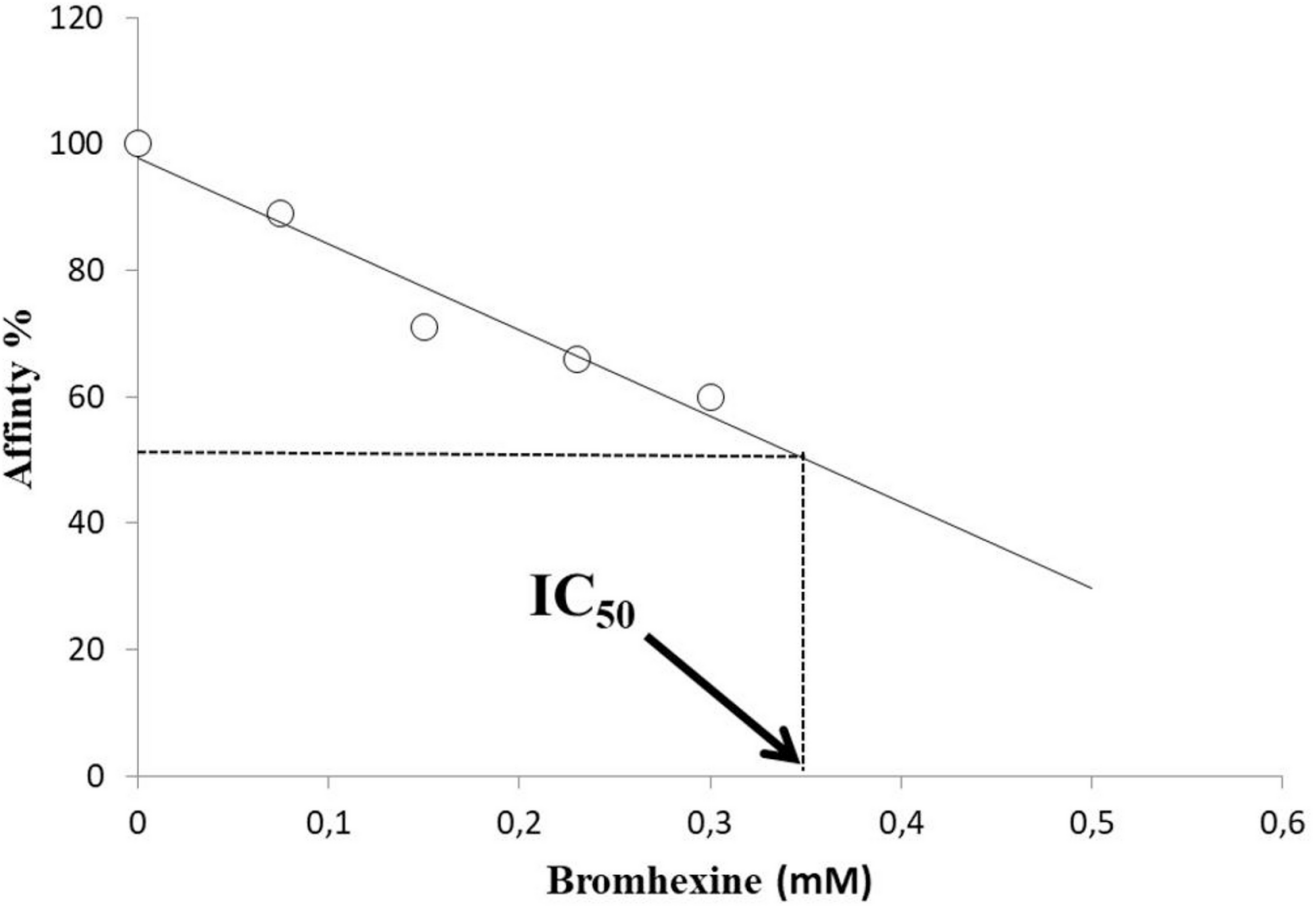
IC_50_ value for Bromhexine in PL. The affinity of the enzyme for its substrate decreased with increasing the drug concentration.

From Fig. 6, different K_m_ values of enzyme were also used to determine the IC_50_ value of Bromhexine. In comparison, various IC_50_ values have been reported for Orlistat, used as reference compound in the current study. The IC_50_ value for Bromhexine against PL was found to be 360 µM (0.36 mM) (Fig. 8).

Different studies have reported IC_50_ values for Orlistat, ranging from 587.70 ± 14.90 µM^36^ to 60.48 ± 0.02 µM^5^. These values indicate that Bromhexine exhibits a similar but possibly milder inhibitory activity against PL. Other tested PL binders reported in the literature are detailed in the supplementary section with their different IC_50_ values and interactions (Table S3).

Considering the multiple gastrointestinal adverse effects associated with Orlistat, which can affect patient compliance, and given that Orlistat is typically prescribed for people with a body mass index (BMI) > 30, or those with a BMI > 27 and concomitant obesity-related risk factors or diseases, it is not suitable for regular use by healthy individuals for weight management. In contrast, repurposing drugs to target PL could offer a viable alternative^1,38^.

A study that closely aligns with our analysis investigated natural products, which are promising for obesity treatment due to their generally lower toxicity and side effects compared to fully synthetic drugs. The IC_50_ values towards PL for two flavonoid compounds, Myricetin and Quercetin, were reported as 337.5 µM and 421.1 µM, respectively. However, in silico studies suggest that the sizes of these molecules and the positioning of hydroxyl groups significantly influence the effectivity of lipase inhibition^39^.

## Conclusions and perspective

Obesity is a chronic metabolic disorder with significant health implications. Given the serious side effects associated with current anti-obesity medications, the search for new therapeutic agents is crucial. This study is the first to investigate Bromhexine, an FDA approved mucolytic drug, as a potential inhibitor of pancreatic lipase. Through a combination of computational modeling and experimental assays, we explored the structural properties and binding mechanisms of Bromhexine, and compared it to the well-established anti-obesity drug Orlistat.

Our findings indicate that Bromhexine inhibits PL through a mixed inhibition mechanism, interacting with residues near or within the enzyme’s active site. In vitro assays corroborated our computational predictions, showing an IC_50_ value of 360 µM and a K_i_ value of 450 µM for Bromhexine. These results suggest that Bromhexine may be a viable alternative for managing obesity with potentially fewer side effects compared to current FDA-approved medications.

While Bromhexine demonstrates promising inhibitory activity against PL, further experimental studies, including in vivo evaluations and physicochemical property assessments, are necessary to fully elucidate its safety profile and therapeutic potential. Based on previous studies^11^, , we plan to extend our investigation by conducting Structure-Activity Relationship (SAR) analyses using Ambroxol, the active metabolite of Bromhexine, and further derivatives thereof. Ambroxol is a widely approved and used mucolytic drug, with a lower side effect profile compared to Orlistat, which makes it an ideal candidate for comparison. The structural difference between Bromhexine and Ambroxol is the additional hydroxyl (−OH) group in Ambroxol, which increases its hydrophilicity. This structural variation provides an opportunity to explore whether hydrophobicity or hydrophilicity are more essential for lipase inhibition. The combination of in silico methods, including molecular docking and MD simulations, and kinetic assays will provide deeper insights into the interactions with the target and help identify the most efficient compound for lipase inhibition, ultimately guiding future therapeutic strategies for obesity management.

## Electronic supplementary material

Below is the link to the electronic supplementary material.

Supplementary Material 1

## Data Availability

Protein structure after preparation (1LPB), docked structures of Bromhexine and Orlistat to PL, and MD simulations trajectories in 3 replicas, are freely available for download at zenodo.org, DOI: 10.5281/zenodo.13983552.
