# Supplementary material for: Combining kinetics and in silico approaches to evaluate bromhexine as an anti-pancreatic lipase agent for obesity management
Source: Sci Rep. 2025 May 26;15:18420. doi: 10.1038/s41598-025-02625-4 (PMC12106798; doi:10.1038/s41598-025-02625-4)
Supplement: Supplementary file 1 — Supplementary Material 1 [file 41598_2025_2625_MOESM1_ESM.pdf]

Combining Kinetics and *in Silico* Approaches to Evaluate Bromhexine as an Anti-Pancreatic Lipase Agent for Obesity Management

Asma Gholami[1,2], Dariush Minai-Tehrani[2], Leif A. Eriksson[1*]*

[1]Department of Chemistry and Molecular Biology, University of Gothenburg, 405 30 Göteborg, Sweden

[2]Bioreserach Lab, Faculty of Life Sciences and Biotechnology, Shahid Beheshti University, Tehran, Iran

*Corresponding authors: leif.eriksson@chem.gu.se, d_mtehrani@sbu.ac.ir

ORCID IDs

AG: 0000-0002-8477-3929

DMT: 0000-0003-3589-7324

LAE: 0000-0001-5654-3109

SUPPORTING INFORMATION

Table S1. Average of RMSD and RMSF values (3 replica) for the PL-Bromhexine complex during 200 ns MD simulations. P3

Figure S1. RMSD and RMSF values (200 ns MD simulations in 3 replica) for the PL- Bromhexine complex. P3

Table S2. Average of RMSD and RMSF values (3 replica) for the PL-Orlistat complex during 200 ns MD simulations. P4

Figure S2. RMSD and RMSF values (200 ns MD simulations in 3 replica) for the PL- Orlistat complex. P4

Table S3. Comparison of active compounds with PL inhibitory activity from various sources, along with their IC_{50} values and interacting amino acid residues, is provided. P5

Figure S3. Comparative interaction analysis of 200 ns MD simulation for Orlistat and Bromhexine, highlighting the types of interactions and their probabilities. P5

References P6

Table S1. Average of RMSD and RMSF values (3 replica) for the PL- Bromhexine complex during 200 ns MD simulations.

	**Average RMSD of Protein (Å)**	**Average RMSD of Ligand (Å)**	**Average RMSF of Protein (Å)**
**Replica 1**	1.61	0.85	0.97
**Replica 2**	1.59	1.52	0.85
**Replica 3**	1.58	1.30	0.77

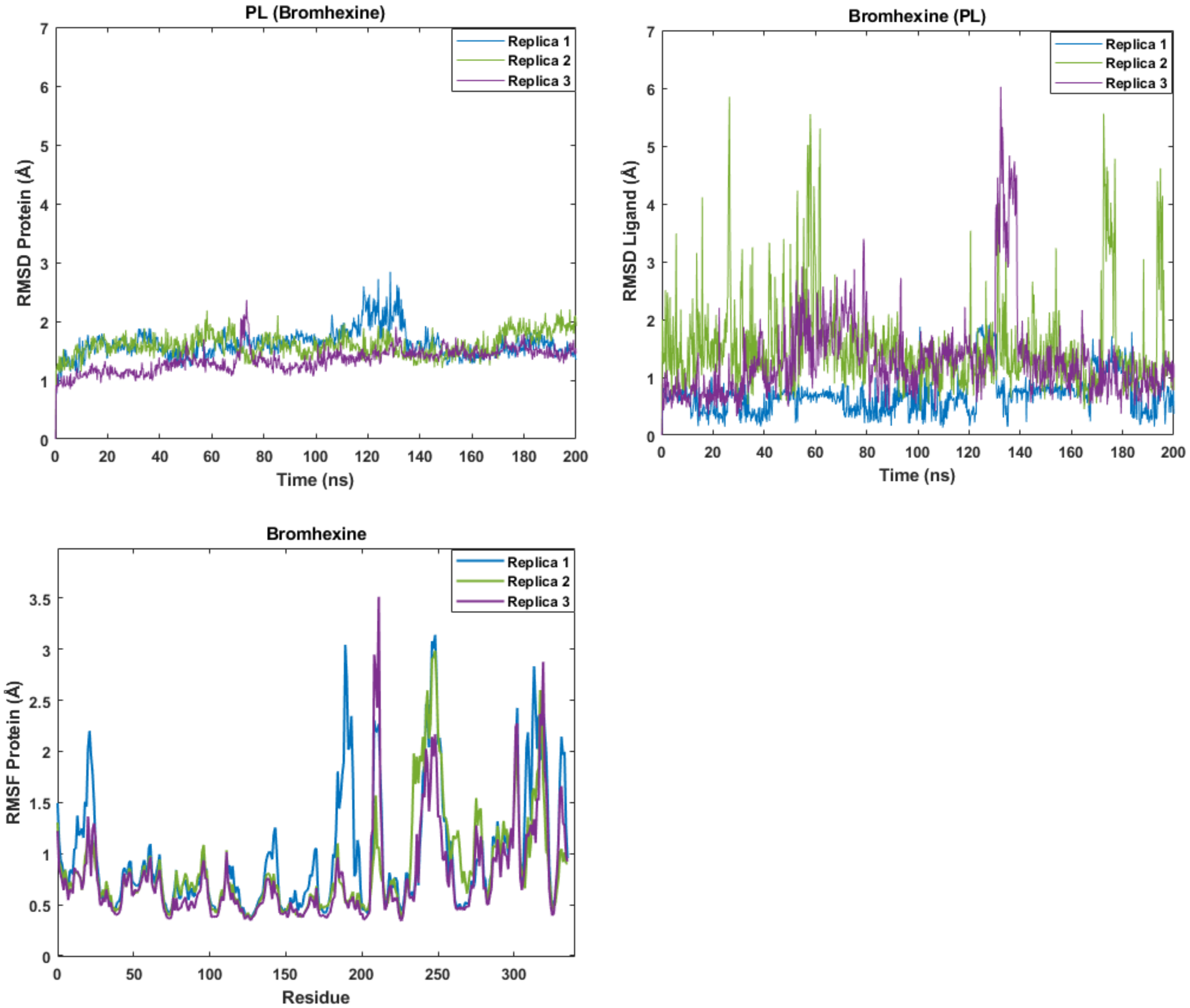

Figure S1. RMSD and RMSF values (200 ns MD simulations in 3 replica) for the PL-Bromhexine complex.

Table S2. Average of RMSD and RMSF values (3 replica) for the PL-Orlistat complex during 200 ns MD simulations.

	Average RMSD of Protein (Å)	Average RMSD of Ligand (Å)	Average RMSF of Protein (Å)
**Replica 1**	1.81	3.00	0.82
**Replica 2**	1.89	2.88	0.80
**Replica 3**	2.52	2.94	0.99

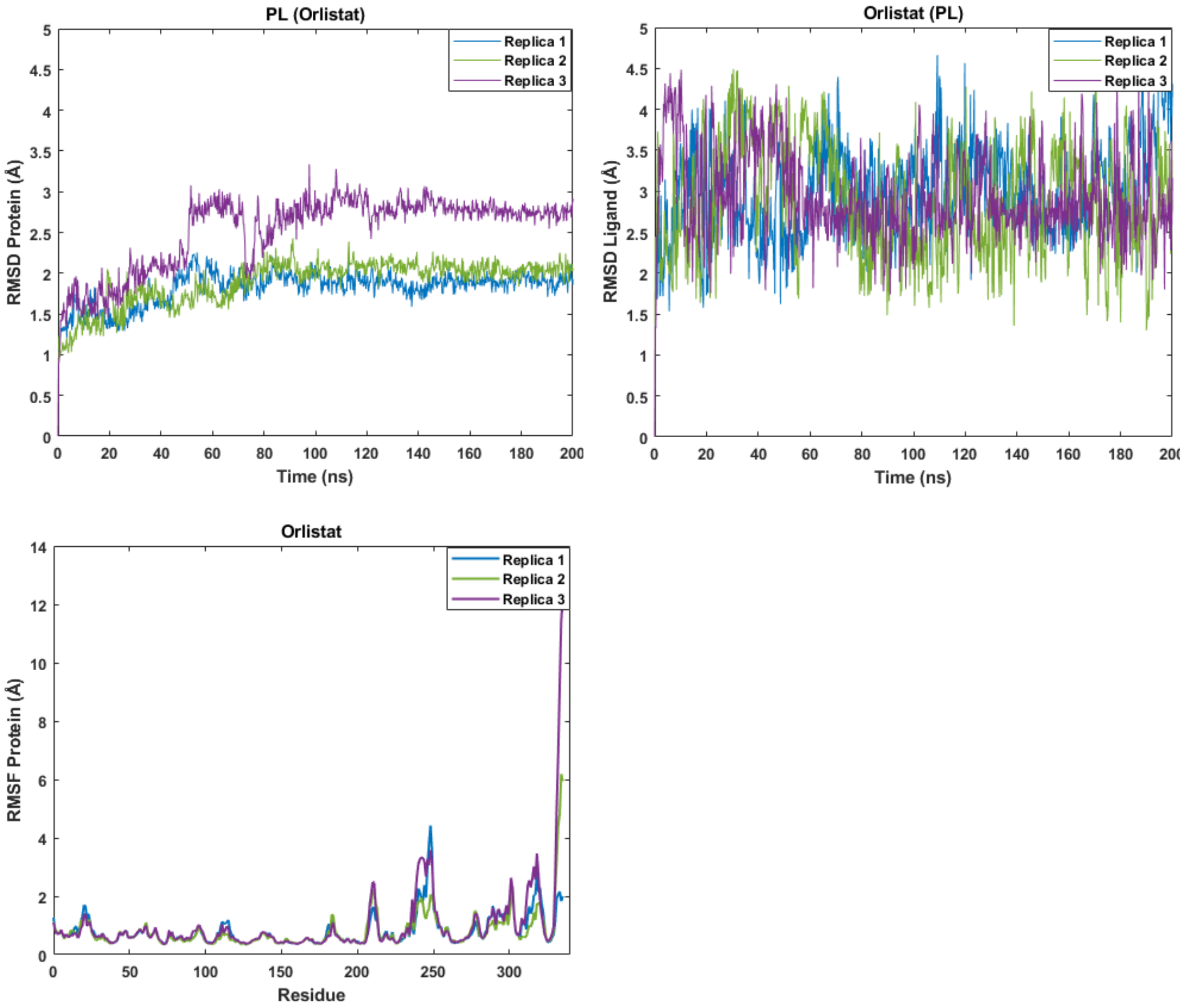

Figure S2. RMSD and RMSF values (200 ns MD simulations in 3 replica) for the PL- Orlistat complex.

Table S3. Comparison of active compounds with PL inhibitory activity from various sources, along with their IC_{50} values and interacting amino acid residues.

Active Compound	IC_{50} (µM)	Residue Interactions	Reference
Chalcone (C82)	1.01	Ser152, Tyr114	(Nguyen et al., 2022)
Indole-thiazolidinedione hybrid analogue (6d)	6.19	Ser152, Phe77, Gly76, His151	(George et al., 2021)
Flavonoid derivatives (F01)	17.68	Ser152, Phe77, Asp79, Arg256	(Tran et al., 2024)
Curcumin	142.24	Ser152, His151, Phe77, Arg256	(Jing et al., 2024)
Ginkgolide B	212.06	Ser152, His263, Tyr 114	(Bustanji et al., 2011)
Berberine (BBR)	315.13	Ser152, His263	(Mohammad et al., 2013)

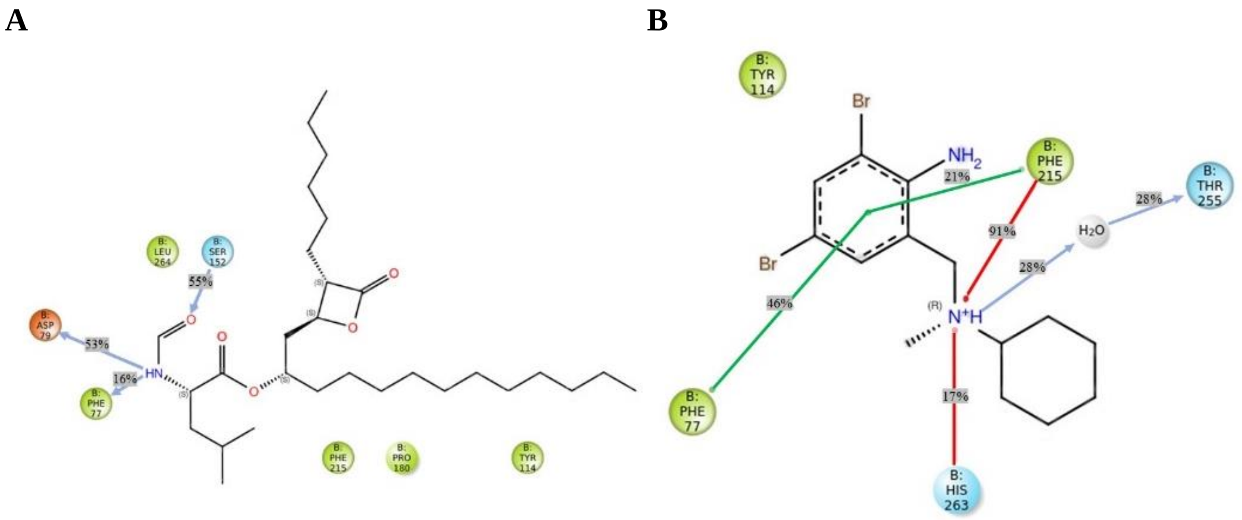

Figure S3. Comparative interaction analysis of 200 ns MD simulation for (A) Orlistat and (B) Bromhexine, highlighting the types of interactions and their probabilities. blue represents hydrogen bonds (H-bonds), red represents π -cation interactions, and green represents π-π stacking interactions.

References

Nguyen, L. C., Le, T. T. A., Dang, T. N., Dao, M. Q., Nguyen, T. H., Vo, M. H., & Tran, T. D. (2022). Natural mimetic 4-benzyloxychalcones as potent pancreatic lipase inhibitors: Virtual screening, synthesis and biological evaluation. *Phytochemistry Letters*, *51*, 28-33.

George, G., Auti, P. S., & Paul, A. T. (2021). Design, synthesis and biological evaluation of N-substituted indole-thiazolidinedione analogues as potential pancreatic lipase inhibitors. *Chemical Biology & Drug Design*, *98*(1), 49-59.

Tran, T. H., Mai, T. T., Ho, T. T. T., Le, T. N. D., Cao, T. C. N., Thai, K. M., & Tran, T. S. (2024). Inhibition of Pancreatic Lipase by Flavonoid Derivatives: In Vitro and In Silico Investigations. *Advances in Pharmacological and Pharmaceutical Sciences*, *2024(1)*, 6655996.

Jing, Y., Luo, L., Zeng, Z., Zhao, X., Huang, R., Song, C., & Jin, S. (2024). Targeted Screening of Curcumin Derivatives as Pancreatic Lipase Inhibitors Using Computer-Aided Drug Design. *ACS Omega*.

Bustanji, Y., Al-Masri, I. M., Mohammad, M., Hudaib, M., Tawaha, K., Tarazi, H., & AlKhatib, H. S. (2011). Pancreatic lipase inhibition activity of trilactone terpenes of Ginkgo biloba. *Journal of Enzyme Inhibition and Medicinal Chemistry*, *26*(4), 453-459.

Mohammad, M., Al-masri, I. M., Issa, A., Khdair, A., & Bustanji, Y. (2013). Inhibition of pancreatic lipase by berberine and dihydroberberine: an investigation by docking simulation and experimental validation. *Medicinal Chemistry Research*, *22*, 2273-2278.
